# Coping Strategies in People Attempting Suicide

**DOI:** 10.5812/ijhrba.16265

**Published:** 2014-03-09

**Authors:** Mohammad-Rafi Bazrafshan, Fereidun Jahangir, Amir Mansouri, Seyyed Hannan Kashfi

**Affiliations:** 1 Student Research Committee, Shiraz University of Medical Sciences, Shiraz, IR Iran; 2 Department of Nursing, Larestan Hazrat Zeinab School of Nursing, Shiraz University of Medical Sciences, Shiraz, IR Iran; 3 Department of Nursing, School of Medical Sciences Gerash, Shiraz University of Medical Sciences, Shiraz, IR Iran

**Keywords:** Adaptation, Psychological, Suicide, Attempted, Educational Status

## Abstract

**Background:**

Having a set of effective coping skills can prevent suicidal behavior by increasing self-control and self-direction. This study examines coping styles used by suicidal patients.

**Objectives:**

The researchers in this study try to identify coping strategies used by suicide attempters admitted to Shiraz Shahid Faghihi Hospital emergency room.

**Materials and Methods:**

This is a analytical cross-sectional study. Participants consisted of 50 suicide-attempted people admitted to Shiraz Faghihi Hospital. Instruments for data collections were a demographic checklist and the coping styles scale of Carver, Schier and Wintrope. Data were collected conveniently and analyzed using descriptive and analytic (Pearson Correlation, Student’s t-tests, and ANOVA) statistical methods.

**Results:**

Suicide attempted people used less useful coping strategies (Mean = 49.32) more than the other strategies (respectively mean of problem focused and emotion focused strategies were 30.27 and 27.83). Using ANOVA, in different educational level, problem focused and less effective coping skills of samples differed significantly (P = 0.009, P = 0.006, respectively). People with low educational level used less effective coping skills. There was a significant difference between men and women scores in use of less effective coping skills (P = 0.029).

**Conclusions:**

Teaching effective coping skills by psychological consultants in suicide attempted people, especially for women and people with low educational level, is important

## 1. Background

Suicide is the conscious act of killing oneself that can be considered a multidimensional unhappiness in a needy person who considers suicide as the best solution for a specific problem (1). Epidemiological studies suggest an increased suicide and attempted suicide incidence in the last two decades in Iran (2). International statistics show that suicide rates in Iran is 9 per 100000 consisting 65% male and 35% female (3). Also, suicide is the cause for 12% of teenagers’ deaths. The rate of successful suicide in young males is 5 times more than the rate of suicide in young girls. But the girls attempt suicide is three times more than boys. The suicide rate among married individuals is 11 per 100,000, while this rate is two folded in single subjects (4). Many studies have been carried out on suicide risk factors. But many people who are exposed to suicide risk factors show no suicidal tendencies. Thus, these people have capabilities which lacks in attempters. Observations show that there may be protective factors in these people that decrease the risk in this population (5). Based on the cognitive theory, suicide attempt results from inability to identify and solve problems when facing them (6). In other words, inability to find solutions for problems and lack of coping strategies to cope with immediate stressors are among characteristics of suicide attempters (4). Coping is the way people deal with and overcome difficulties. Coping skills are methods available for individual in doing each action. Having an efficient collection of coping skills strengthens individual's sense of self-control and self-direction. But when a person's vulnerability is high, the individual shows non-adaptive behavior even in times of mild stresses. However, the higher the coping resources of people, the lower the possibility of getting caught in situations they are vulnerable (7). There are two types of coping skills when facing stress:

1. Action-based coping skills include dealing directly with the cause of stress like finding a job for a person with financial problems and studying to prepare for exams; and
2. emotional based coping skills decrease the stress symptoms without addressing the main sources of stress.

Crying, sleeping or discussing about stress with a friend are among emotional based coping strategies. These kinds of skills improve the person's feelings, but will not ultimately lead to solution of problems. Thus, it seems that action-based coping skills are superior to emotional based ones; because they directly reduce the sources of stress (8). However, some of the behaviors associated with emotional based and avoidant coping strategies such as self-deprecation and lack of involvement in the issue are associated with depression and suicidal ideas (9). Based on the Educational Theory, for people who have attempted suicide, interventions should focus on training methods that lead them to decreased unpleasant and increased pleasant incidents. One of these techniques is to use adapting methods. To use these techniques, first, the kind of coping skills, which these people use when facing stressful life events, should be identified (6).

## 2. Objectives

Conducting a research to identify coping strategies in people who have attempted suicide is necessary. Furthermore, by identifying the suicide risk factors in people who have attempted suicide, researchers can recognize suicide predictors and introduce more appropriate interventions to prevent the problem. Therefore, the researchers in this study try to identify coping strategies, used by suicide attempters, admitted to Shiraz Shahid Faghihi Hospital emergency room.

## 3. Materials and Methods 

In this cross-sectional study, the samples consisted of patient admitted to Shiraz Shahid Faghihi Hospital emergency room. The sample size of 50 subjects were selected by the formula for determining the sample size in cross-sectional studies (z^2^σ^2^/d^2^), considering 90% confidence level, 20% error and variance obtained from similar studies (0.70). They were selected through convenient sampling. Initially, suicide attempters were identified. Next, using questionnaires, they were investigated in terms of coping strategies they employed.

The data collection instrument was Carver, Scheier, and Weintraub cope inventory (10). The cope inventory includes 18 coping scales with four-point Likert scale (1 = I usually don’t do this at all, 2 = I usually do this a little bit, 3 = I usually do this a medium amount, 4 = I usually do this a lot) assessed with 52 items deal-with option. The scales are classified in three groups.

1. problem-focused coping (active coping, planning, suppression of competing activities, restraint coping, seeking of instrumental social support);
2. emotional-focused coping (seeking of emotional social support, positive reinterpretation, acceptance, denial, turning to religion); and
3. scales that measure coping responses that are less useful (focus on and venting of emotions, behavioral disengagement, mental disengagement, impulsiveness, superstitious thinking, negative thinking, wishful thinking, and use of tobacco and drugs (10).

To determine the reliability of the instrument, the internal consistency method was used. In a pilot study, questionnaires were distributed among 24 of the subjects and the alpha coefficient was calculated using SPSS that was 0.78. Data were analyzed by SPSS. Descriptive and statistical analyses were used for data analysis.

## 4. Results

The research subjects were mostly in the age group 10-20 (46.74%) and 21-30 (42.39%). The mean age of subjects was 23.48 ± 7.95 years, consisted of 56.52% females, 30.43% were married and 69.57% were single. In terms of education level, most had junior high school (40.40%) and high school (39.13%) degrees. The mean scores for problem-focused, emotional focuses and less useful coping strategies were 30.27, 27.83 and 49.32, respectively (Table 1). The relationship between coping strategies and age was investigated using Pearson's correlation coefficient. No significant relationship was found in any of the areas (P > 0.05) (Table 2). The relationship between coping strategies and marital status was investigated with t-test. No significant relationship was found in any of these areas (P > 0.05) (Table 3). The relationship between coping strategies and gender was investigated with t-test. A significant relationship was found between the mean scores of the two groups in the area of less useful coping strategies (P < 0.05) (Table 4). The relationship between coping strategies and education was investigated with one-way ANOVA test. Given the mean scores on different research areas, it was found that people who had a higher education level mostly used problem focused coping methods and used less useful methods the least (P < 0.05) (Table 5). As we had ANOVA results for problem focused and less useful coping methods, for determining the educational level groups with different scores on these components, multiple comparison tests must be used. Bonferroni's multiple comparison tests was used for this purpose. Results in Table 6 show that the only significant difference in this area is between elementary school and college degrees (P < 0.05).

**Table 1.** Coping Methods Used by Suicide Attempters Admitted to Shahid Faghihi Hospital Affiliated With Shiraz University of Medical Sciences

	Mean ± SD	Total Mean	Total SD
**Problem-focused coping**		30.27	4.96
Active coping	6.02 ± 1.59		
Planning	5.57 ± 1.73		
Suppression of competing activities	6.93 ± 0.93		
Restraint coping	6.12 ± 0.93		
Seeking of instrumental social support	5.74 ± 1.83		
**Emotion-focused**		27.83	5.60
Seeking of emotional social support	5.30 ± 1.83		
Positive reinterpretation	7 ± 1.22		
Acceptance	5.27 ± 1.25		
Denial	6.80 ± 1.56		
Turning to religion	6.73 ± 1.02		
**Less useful methods**		49.32	4.84
Focus on emotion and its expression	5.28 ± 1.13		
Mental disengagement	5.80 ± 2.06		
Behavioral disengagement	6.35 ± 1.78		
Impulsiveness	5.33 ± 1.33		
Superstitious thinking	6.33 ± 1.34		
Negative thinking	6 ± 2.28		
Wishful thinking	5.50 ± 1.35		
Use of tobacco and drug	6.11 ± 1.65		

**Table 2.** Correlations Between Coping Strategies and Age of Suicide Attempters Admitted to Shahid Faghihi Hospital Affiliated With Shiraz University of Medical Sciences

	Mean ± SD	Significance Level	Correlation Coefficient
**Problem focused**	30.27 ± 4.96	0.789	0.043
**Emotion focused**	27.83 ± 5.60	0.853	- 0.028
**Less useful**	49.32 ± 4.84	0.945	0.011

**Table 3.** Comparison of Mean Scores of Coping Strategies According to Marital Status, Gender and Education Level of Suicide Attempters Admitted to Shahid Faghihi Hospital Affiliated With Shiraz University of Medical Sciences^a^

	Problem-Focused	Emotion-Focused	Less Useful
**Marital status**			
Married	31.44 ± 3.79	27.06 ± 12.8	49.94 ± 3.82
Single	30.74 ± 3.91	29.62 ± 6.69	50 ± 4.51
P	0.553	0.254	0.962
**Gender**			
Female	30.54 ± 3.25	28.95 ± 7.63	51.45 ± 4.35
Male	31.5 ± 4.52	28.68 ± 7.23	48.82 ± 3.88
P	0.385	0.896	0.029
**Education level**			
Elementary	29.75 ± 2.63	31.20 ± 7.63	57.21 ± 1.10
Junior high school	30.55 ± 3.78	31 ± 0.24	50.60 ± 3.95
Diploma	34 ± 5.33	29.75 ± 6.93	49.75 ± 4.50
College	35 ± 0.02	27 ± 4.48	48.75 ± 4.08
P	0.009	0.202	0.006
F	4.58	0.877	3.22

^a^ Data are present as Mean ± SD. Significant at P < 0.05.

**Table 4.** Comparison of Mean Scores of Coping Strategies and Gender of Suicide Attempters Admitted to Shahid Faghihi Hospital Affiliated With Shiraz University of Medical Sciences^a^

Coping Strategies	Problem-focused	Emotion-focused	Less Useful
**Gender**			
Female	30.54 ± 3.25	28.95 ± 7.63	51.45 ± 4.35
Male	31.5 ± 4.52	28.68 ± 7.23	48.82 ± 3.88
P value	0.385	0.896	0.029

^a^ Significant at P < 0.05

**Table 5.** Comparison of Mean scores of Coping Strategies in Individuals With Education Level of Suicide Attempters Admitted to Shahid Faghihi Hospital Affiliated With Shiraz University of Medical Sciences ^a^

Coping Strategies	Problem-focused	Emotion-focused	Less Useful
**Education level**			
Elementary	29.75 ± 2.63	31.20 ± 7.63	57.21 ± 1.10
Junior high school	30.55 ± 3.78	31 ± 0.24	50.60 ± 3.95
Diploma	34 ± 5.33	29.75 ± 6.93	49.75 ± 4.50
College	35 ± 0.02	27 ± 4.48	48.75 ± 4.08
**P value**	0.009	0.202	0.006
**F value**	4.58	0.877	3.22

^a^ Significant at P < 0.05.

**Table 6.** Comparison of Mean Scores of Coping Strategies in Individuals With Education Level of Suicide Attempters Admitted to Shahid Faghihi Hospital Affiliated With Shiraz University of Medical Sciences^a^

	Education Level (I)	Mean Difference (I-J)	Standard Error	P value
**Problem focused**				
Elementary				
	junior high school	- 0.80	3.27	0.624
	diploma	- 4.25	3.28	0.059
	college	- 5.25	3.63	0.033
Junior high school				
	diploma	- 3.45	1.61	0.215
	college	- 4.45	2.24	0.141
Diploma				
	college	- 1	2.26	1
**Less useful**				
Elementary				
	junior high school	6.61	2.72	1
	diploma	7.46	2.70	0.179
	college	8.46	3.12	0.048
Junior high school				
	diploma	0.85	1.59	0.210
	college	1.85	2.22	0.055
Diploma				
	college	1	2.21	1

^a^ Significant at P < 0.05.

## 5. Discussion

The results of this study show that suicide attempted people used less useful coping strategies (mean = 49.32) more than the other strategies similarly. In a survey conducted in 2007, Andover et al. showed that students who committed self-injury used less useful defense techniques more than others (11). In a study conducted on 3127 school students by Sun et al., the most important risk factor for suicide attempt was negative adaptive strategies (12). 

In the present study mean score for problem focused strategies (Mean = 30.27) was higher than emotion focused strategies (Mean = 27.83). Chapman et al. found that there was a significant correlation between history of suicide attempts and problem-focused coping strategies in female prisoners. Women who had a history of suicide attempts used problem-focused coping strategies less than others (13). In another study conducted on 6020 15 to 16-year-old students, Evans et al. concluded that students who had a history of self-injury, showed higher use of avoidance behaviors and less focus on problem-focused ones (14). In the subjects included in a study by Degraff on people with spinal cord injuries, people who were suffering from spinal cord injuries used emotion-focused coping strategies, which were their best defense mechanisms against stress (15). In spite of divergence in using coping mechanism by afflicted people, having broader set of mechanisms is useful in adapting to stress. For example, emotion-focused coping strategies do not directly remove the source of stress; however, they reduce the symptoms. Therefore, health professionals can teach such strategies to at-risk people to prevent suicide.

Ineffective or less useful strategies used by female suicide attempters were significantly higher than men (P = 0.029). The mean score of women in these coping strategies were higher. Several studies have shown that defensive techniques used by men and women to deal with problems are different. It is generally agreed upon that women use emotion-focused and avoidance coping strategies and techniques to deal with problems, while men use problem-focused ones (16).

No significant relationship was found between age, marital status and coping strategies used by individuals (P > 0.05). Kim and Agrusa found that age does not affect coping strategies to deal with problems and adaptive methods older people use to deal with problems are similar to those used by young people (16). Significant difference was found between level of education and type of coping strategies used i.e. problem-focused and less effective coping strategies. Results of multiple comparison tests show that people who have college education use more problem-focused coping strategies than those who are less educated. The results of the latest research in 2012 show that compared with people who have died of natural causes (15-64 years old people) victims of suicide in both genders were mostly people with low educational levels. People with lower educational achievement were more likely to commit suicide in face of general failure and Stigma (17). In a study by Sun et al. conducted on 3127 high school students, school life satisfaction acted as a protective factor against suicidal thoughts and actions (12) and Kim and Agrusa stated that people of higher social class (higher education and income) are more likely to use problem-focused adaptive strategies (16). In a study on people who had attempted suicide, Saberi-Zafarghandi indicated that only 10.2% of subjects had academic education (1). However, most of the subjects in this study had high school education (43.4%) and the illiterate subjects accounted only 2.5%. The researchers inferred that higher level of education increases the individual's ability to efficiently deal with problems; however it may also act as a stressor.

Considering that most subjects used ineffective coping strategies to deal with problems, the importance of teaching effective coping strategies, especially to vulnerable groups in the society by consultants, is evident. People who are less educated mostly use less useful coping strategies. Thus, teaching effective coping strategies to people with lower educational levels is of particular importance. For those who have a higher education level and are involved in education related issues (such as university students), teaching strategies to deal with educational problems and counseling can be effective. In this study, significant differences were found between gender and less useful non-adaptive methods. It is generally agreed upon that women mostly use emotion-focused coping strategies, but men tend to use problem-focused coping strategies to deal with their problems (16). Nonetheless, other effective factors like cultural barriers should not be ignored. Perhaps, besides individual differences, one reason why women mostly use less useful strategies is that they face barriers when they want to use effective strategies, even in Western countries. Identification of these barriers can be an effective measure for preventing suicide in women. Therefore, further research is suggested to determine the reasons for this difference. One of the limitations of this study was that the study primarily was done to determine the coping strategies of suicide attempted patients admitted to hospital. While there are many people who are having suicidal thoughts without admission in hospital. Therefore, studies on this group of people for identification of the coping strategies are required.

Low sample size was another limitation of the study. Because we only included the subjects who were admitted to Shahid Faghihi Hospital. We assume that to determine the educational needs of people who attempt suicide or with suicidal thoughts, more samples are required. To be due to individual, ethnicity, religion, cultural, etc. differences, it used widely as a society and its subsets such as schools, universities, hospitals.

## References

[1] Saberi-Zafarghandi MB, Ghorbani R, Mousavi S (2005). Epidemiologic study on suicide attempt in affiliated hospitals of Semnan University of medical sciences.. J Semnan Univ Med Sci..

[2] Koushan M, Shegarf Nakhaee MR, Rabbanizadeh A, Heidari A, Tofighian T, Maskani K (2008). Study of the Risk Factors in Suicide Cases Admitted to Vase'ee Emergency Clinic in Sabzevar, Iran .. J Sabzevar Univ Med Sci..

[3] Mohammadi G, Saadati A (2004). Survey of epidemiology and ethiology of suicidal attemt and relation tosociodemographic factors in the adminstrated emergency unit central hospital of neishabur in 2003.. J Fundam Mental Health..

[4] Esmaeilnia T, Faramarzi M, Mousavi M, Shamsi G (2005). Causes of attempted suicide among women of Babol town (2001-2002).. J Babol Univ Med Sci..

[5] Beautrais A (2003). Suicide in New Zealand II: a review of risk factors and prevention.. J N Z Med Ass..

[6] Fortinash KM, Holody W, Patricia A (2004). Psychiatric mental health nursing..

[7] Jahangir F, Bazrafshan MR, Zangouei AR, Raeisi T (2009). Comparsion of coping mechanisms used by sucidal attempt patients and those without suicidal history.. Med J Hormozgan Univ..

[8] Veenema AH, Meijer OC, De Kloet ER, Koolhaas JM (2003). Genetic selection for coping style predicts stressor susceptibility.. J Neuroendocrinol..

[9] Horwitz AG, Hill RM, King CA (2011). Specific coping behaviors in relation to adolescent depression and suicidal ideation.. J adolesc..

[10] Carver CS, Scheier MF, Weintraub JK (1989). Assessing coping strategies: a theoretically based approach.. J Pers Soci Psych..

[11] Andover MS, Peter CM, Gibb BE (2007). Self-mutilation and coping strategies in a college sample.. Suicide Life Threat Behav..

[12] Sun Y, Tao FB, Gao M (2006). Suicidal behaviors and correlated psychological factors in secondary school students.. J Zhonghua Liu Xing Bing Xue Za Zhi.

[13] Chapman A, Specht MW, Cellucci T (2005). Factors associated with suicide attempts in female inmates: the hegemony of hopelessness.. Suicide Life Threat Behav..

[14] Evans E, Hawton K, Rodham K (2005). In what ways are adolescents who engage in self-harm or experience thoughts of self-harm different in terms of help-seeking, communication and coping strategies?. J Adolesc ..

[15] Degraff AH, Schaffer J (2008). Emotion-focused coping: a primary defense against stress for people Living with spinal cord injury.. J Rehabil..

[16] Kim HJ, Agrusa J (2010). Emotional intelligence and coping styles among hospitality industry employees.. International CHRIE conference-refereed track..

[17] Pompili M, Vichi M, Qin P, Innamorati M, DeLeo D, Girardi P (2013). Does the level of education influence completed suicide? A nationwide register study.. J Affect Disord..

